# Supplementary material for: Patterns of Intron Gain and Loss in Fungi
Source: PLoS Biol. 2004 Nov 30;2(12):e422. doi: 10.1371/journal.pbio.0020422 (PMC532390; doi:10.1371/journal.pbio.0020422)
Supplement: Table S1 — Also available at http://genes.mit.edu/NielsenEtAl/. (4.3 MB ZIP). [file pbio.0020422.st001.zip › NielsenEtAl/html/666.html]

AN1996.1.NCU08370.1.MG03735.1.FG01498.1


```
 CLUSTAL W (1.82) Multiple Sequence Alignments - Introns Inserted


Sequence 1: NCU08370.1	630 aa
Sequence 2: MG03735.1	614 aa
Sequence 3: FG01498.1	629 aa
Sequence 4: AN1996.1	655 aa
Alignment Length: 716 aa
Number Identitical Residues: 242 aa
Alignment Score (without introns) 11766


MG03735.1 	--MSKRG-------PSVL0VTDARDRPSARGSASRGAPARAGQGGAGRDKMPMRFVSVDN
NCU08370.1	MASAKRG-------PSVL0VTDARQRPSPPSAPRRMGPT----GGPGR-LLNGRSFTVDN
FG01498.1 	-MSSKRG-------PSVL0VTDARQRQPQRRVLQRQDTG---AGAPGS-QAPMRFMTVDN
AN1996.1  	-MSSSRGRGPNQRSPHVL0VTDSREQHSSAQPRRRRSPA------------ATRFITVDN
          	   :.** .... :* ** ***:*:: .      *  .               * .:***

MG03735.1 	VLQYASEIPSGQPRVPPPGQQG--------AHLHHARPGGGPSTRRNSHG-GLPTLASIR
NCU08370.1	VLQYSSDIPSGLPRLPP-GQ----------------RP-TRAAMRRSSHGTGLPN-ASAR
FG01498.1 	VLQYNSQIPSGQPRGPPGPATL--------PSGRHPGPPGSALSRRVSSG-GLPN-SQSR
AN1996.1  	VLQYASDIPSMQQRNPPDAARLRQRPRLPSATGGLIGSGAGGASRNATSSANASSTAAAI
          	**** *:***   * **      . .  .:.      .      *. : .:. .. :   

MG03735.1 	---TGLQNVPSRTTKISEKLVLLPEM--EKGDEDEDEEDADGES-----LPPPVLRQDSS
NCU08370.1	---TGQQIVPSRTTKVSEKLVLLPET--PDNEDALGDEELERR--------ASILRASED
FG01498.1 	---TGQQ-IPSRTTKISEKLVLLPEA--EDNGDDVDEEIESES--------ILARRVQDD
AN1996.1  	GRLAAQAHLPPRTTKVSEKLVLLPEEGVEPQPEDEDEEEEEQGLGDEIVDEELVQRIARD
          	.  :.   :*.****:********* .     :  .:*  .   ...        *   .

MG03735.1 	EDRPLRDEELDVLRKRGGIRG----------------------------KSFAERLPKVQ
NCU08370.1	DNRPLKDEELDVLRKRGGLRG----------------------------KSYAERLPKIQ
FG01498.1 	ENRPLKDEELDVLKKRGGVRG----------------------------KSFAERLSKTQ
AN1996.1  	KNIDPDSVRHTLLAQKKKLRGDFSVDNDVAPLLAEEDRTRKRAIAPERAKSYAERLPKAR
          	.:    . .  :* ::  :**. : ... :.  :... : . : :.. :**:****.* :

MG03735.1 	RGDRVSRLTAYCTAQAVKMKPTADFLRTKLGAKTKLYDDCLYTVFHLPLLPGTDGYRVRS
NCU08370.1	RGEKVSRLTAYCTAQAYKMQATAEFLKKKHEAKTKLYDDCLYTVYHLPLLPGVDGYRLRS
FG01498.1 	RTDKVSRLTAYCTAQAYKIKPTAEFLRKKHEAKTKIYDDCLYVIYALPLLNGNDGTRIRS
AN1996.1  	RAEKLARVTAYCTAQAYKMSSLSSFVKDTHGGRTKLYDDCLYTAYHLPLLPGHEGYRVRS
          	* ::::*:******** *:.. :.*:: .  .:**:******. : **** * :* *:**

MG03735.1 	RPVLKTPGTGKTVLDIEIERSERRDEHMGYFYEYPT------------------TEEGID
NCU08370.1	RPILKTPGTGKTVLDLEIERSERRDEHEGYWDEYPFNMHGSADSPSNGTTLLQHAHSGVD
FG01498.1 	RPILKTPGTGKTVLDLEIERSEQRDHHEGYFDDDAYEHPS--------------PDRGHE
AN1996.1  	SPLVKKPG-GKSLLDEEIERNELRDHDEDYIESEEHSILEGR----------PDEHENQE
          	 *::*.** **::** ****.* **.. .*  .   .   .              . . :

MG03735.1 	VSAPVNGSHD-MAMNGP-------SPVDRLVAEAKSFAEMFVFSYGVVVFWNFTENQEKD
NCU08370.1	ETGAHSSDHDQKTLDHPP-----ISPINRLAPDAKRFGEMFVFSYGVVVFWNFTEHQEKD
FG01498.1 	IPARFTDRPSTPERSNPFHHDDDVSSMNRLAPDAKNFAEMFVYSYGVVVFWNFTEHQEKD
AN1996.1  	RNGRPSSSKGTANDGHSQ----ILSPGSTPARLPYDVAEMFVFSYGVVVFWNFTAKQERD
          	  .  ..  .    . .       *. .  .  .  ..****:*********** :**:*

MG03735.1 	ILADMTFSENEE----GLSLQTRPLDQEDYETEEFHFDYSADVKRPRVFNDMITLLPRSD
NCU08370.1	ILADLTFAENET----GVSLATRPLDQNDFETEEFHFEYSADVDRPRVFNDMITLLPRSD
FG01498.1 	ILADLTFADADAVENGATSLLTRPLDQEDYETEEFHFEYSADIKRPRIFNDMITLLPKSD
AN1996.1  	ILADLAFATSSATG-SPITLATLPLQEEDFETEEFHFEYSTEISRPRVYNDMITLRS-GD
          	****::*:  .    .  :* * **:::*:*******:**:::.***::****** . .*

MG03735.1 	HMVKLTISHAIAQSTKLCYFEEKMGETMLDAQHVPKQLALTGELAMTRTEIVKILGRLFK
NCU08370.1	HMVKLTISHAIAQSTKLCFFEERMSETMEGAQHVPKRLALTGKLDMDRTEVLKILGQLFK
FG01498.1 	HMIKLTISHAIAQSTRLCFFEERMSETMLDAQHVPKMLALTGELKMTRTEIVRMLGKLFR
AN1996.1  	HMIKLAISHGIAQSTKLCFFEEVMARQMAEAKDVPRRLAMTGKLGLKREEVFRILGKLFK
          	**:**:***.*****:**:*** *.. *  *:.**: **:**:* : * *:.::**:**:

MG03735.1 	SRVDINLS1SNILDVPNFFWDSEPTLHPLYVAIRDYLEIDQRIKVLNERCRVFLDLAEIL
NCU08370.1	SRVDINLS1SNILEKPNFFWDSEPTLGPLYEAIRDYLEIETRTRVLNERCQVFLDLASIL
FG01498.1 	SRVDINLS1SNILDVPNFFWESEPTLHPLYAAIREYLEIDPRIKVLNERCRVFLDLAEIL
AN1996.1  	SRVEVNLS1SNVLDVPNFFWESEPTLYPLYIAVREYLEIKPRIQVLNERCRVFLDLAEIL
          	***::*** **:*: *****:***** *** *:*:****. * :******:******.**

MG03735.1 	SDSVADAKMSS~ITWIIIILIVVSILVTVTEVTLRFGILEKERGKKGVGD----EGGQPE
NCU08370.1	SDHVADAKMSY~ITWIIIILIVISIMVTVTEVLLRFAILSNEKAKDGANGPANVIGGGNL
FG01498.1 	SDSVADAKMSY~ITWIIIVLIILSIMVTTAEVGIRFGMLNKAKGTN-VNR----IITAPV
AN1996.1  	SDSIADSNTSH1QTWIIIVLIVISILVTTSEVFLRFGLLNSGKGTSAAGFG---------
          	** :**:: *   *****:**::**:**.:** :**.:*.. :......           

MG03735.1 	LKSD-------LLSRLRDANISLDELRIWGQGLSEPEKGAVCGSTYVGRTFAGA-----
NCU08370.1	LTAHSRIDLRSLESKLANANLTLDDLRLWSAAMNEQEKKSICGAEIVGTTFKGL-----
FG01498.1 	LGHG----MEQTVLPPSDLEILAQTLGLQANASFEDVHKSIWALQGKDLPNASILHEDI
AN1996.1  	------------------AALFGKVIRGFSSTTRTCSCPDVMGSSSNFSGLDGFS----
          	                    :  . :   .          : .         .
```
